# Prevalence and prognosis of patients with breast cancer eligible for adjuvant abemaciclib or ribociclib: a nationwide population-based study

**DOI:** 10.1016/j.lanepe.2025.101471

**Published:** 2025-09-29

**Authors:** Xingrong Liu, Behnaz Binicy, Balazs Acs, Louise Eriksson Bergman, Sibylle Loibl, Michael Gnant, Michael Untch, Antonios Valachis, Jonas Bergh, Johan Hartman, Theodoros Foukakis, Alexios Matikas

**Affiliations:** a Department of Oncology/Pathology, Karolinska Institutet, Stockholm, Sweden; b Department of Clinical Pathology and Cancer Diagnostics, Karolinska University Hospital, Stockholm, Sweden; c German Breast Group, Neu-Isenburg, Germany; d Comprehensive Cancer Center, Medical University of Vienna, Vienna, Austria; e Austrian Breast and Colorectal Cancer Study Group, Vienna, Austria; f Helios Klinikum Berlin-Buch, Berlin, Germany; g Department of Oncology, Faculty of Medicine and Health, Örebro University, Örebro, Sweden; h Breast Center, Karolinska Comprehensive Cancer Center, Stockholm, Sweden

**Keywords:** Abemaciclib, Adjuvant, Breast cancer, CDK4/6 inhibitors, monarchE, NATALEE, Ribociclib

## Abstract

**Background:**

Nationwide studies on the prevalence, characteristics and long-term prognosis of patients with early breast cancer and a potential indication for adjuvant CDK4/6 inhibitors are lacking.

**Methods:**

We leveraged a prospectively collected nationwide cohort of all patients treated for breast cancer in Sweden in 2007–2023 and identified patients fulfilling the monarchE and/or the NATALEE eligibility criteria, to estimate the proportion of eligible patients for adjuvant CDK4/6 inhibitors and their outcomes. Overall survival (OS) was the primary endpoint of interest. In addition, we compared distant relapse-free survival and OS of a patient subset treated in the Stockholm–Gotland region in 2012–2020 to patients enrolled in the phase 3 PANTHER trial (NCT00798070) in 2007–2011, before and after alignment of key inclusion criteria.

**Findings:**

Of 52,602 patients with ER-positive, HER2-negative breast cancer, 9553 (18.2%) fulfilled both the monarchE and the NATALEE criteria, 9800 (18.6%) fulfilled the NATALEE criteria only and 745 patients (1.4%) fulfilled the monarchE criteria only. Median follow-up was 6.87 years. Ten-year OS rates were 65.7% (95% CI 65.3%–67.0%) for monarchE-eligible and 70.2% (95% CI 69.3%–71.1%) for NATALEE-eligible patients, and 84.0% (95% CI 83.4%–84.6%) for non-eligible, 75.5% (74.4$–76.7%) for NATALEE-only eligible and 64.3% (95% CI 62.9%–65.7%) for concordant eligible patients for both studies (p < 0.0001). Before alignment of inclusion criteria, patients treated within the PANTHER trial had improved outcomes compared to those treated in clinical routine. However, the differences in outcomes disappeared after alignment.

**Interpretation:**

These results inform on the prevalence and prognosis of candidates for adjuvant CDK4/6 inhibitors at a nationwide level.

**Funding:**

10.13039/501100002794Cancerfonden, Bröstcancerförbundet, 10.13039/501100007232Radiumhemmets Forskningsfonder, Amgen, Roche, Sanofi-Aventis.

**Research in context**

**Evidence before this study**

Following the regulatory approvals of adjuvant abemaciclib and ribociclib for intermediate and high-risk luminal breast cancer, several real-world studies have investigated the prognosis of patients that would be eligible for the corresponding monarchE and NATALEE trials. We performed a literature search of the Medline and Embase databases in May 12th 2025 for relevant studies, using the terms “breast cancer”, “adjuvant”, “CDK4/6 inhibitors”, “monarchE”, “abemaciclib”, “NATALEE”, “ribociclib”. Reported prevalence and patient outcomes vary widely, depending on the data source (single institution, population-based or clinical trial cohort). In our search we could not identify any nationwide studies with complete coverage that would minimize selection bias.

**Added value of this study**

With this large nationwide cohort study, we amend the limitations of prior studies on the subject and provide more accurate estimates of the prevalence and long-term survival of patients fulfilling the eligibility criteria of the monarchE and NATALEE trials. In addition, we compare for the first time outcomes of these high-risk patients treated in a phase 3 trial compared to clinical routine and highlight the importance of appropriate alignment of inclusion criteria to avoid pitfalls in interpreting survival analyses from selected trial populations.

**Implications of all the available evidence**

Real world studies on patients eligible for monarchE and NATALEE highlight the efficacy-effectiveness gap with the pivotal trials, underscore the relatively poor survival outcomes in this high-risk population and provide estimates of the proportion of patients potentially eligible for ribociclib or abemaciclib, so that healthcare systems can better plan for the implementation and resource allocation of these therapies.

## Introduction

Owing to its long natural history, hormone receptor (ER) positive and human epidermal growth factor receptor 2 (HER2) negative breast cancer carries a guarded prognosis. Despite adjuvant systemic therapy with chemotherapy and endocrine treatment, the annual rate of relapse is stable over an extended period, leading to substantial cumulative risk of metastatic recurrence and death.^1^ Early detection thanks to organized population screening and advances in adjuvant chemotherapy,^2^ radiotherapy,^3^ and endocrine therapy^4^, ^5^, ^6^ have improved outcomes.^7^ However, long-term recurrence risk remains even for patients that are relapse-free at least ten years postoperatively.^8^

Following the clinically meaningful improvement of outcomes in the palliative setting with the addition of cyclin dependent kinases 4/6 (CDK4/6) inhibitors to endocrine treatment, all three approved CDK4/6 inhibitors were evaluated as postoperative therapy.^6^ While the two trials investigating palbociclib, PALLAS and PENELOPE-B,^9^^,^^10^ were negative for the primary endpoint, monarchE (abemaciclib) and NATALEE (ribociclib) reported improved disease-free and distant disease-free survival with combination therapy compared to endocrine treatment alone, following adjuvant chemotherapy for most patients.^11^^,^^12^ These results have led to regulatory approval, among others by the European Medicines Agency and the United States Food and Drug Administration, for the treatment of patients with intermediate and high-risk breast cancer according to the inclusion criteria of the respective trials with abemaciclib for two years or ribociclib for three years.^13^ In addition, the two trials highlighted the guarded prognosis of enrolled patients treated with chemotherapy and endocrine treatment alone, as supportive evidence for the use of adjuvant CDK4/6 inhibitors.^14^ How the inclusion criteria are reflected at the population level in terms of prevalence and prognosis compared to within a clinical trial is however unclear. The latter is of particular interest, since published studies mainly comprise post hoc analyses of clinical trial cohorts,^15^^,^^16^ which due to specific inclusion and exclusion criteria might be susceptible to selection bias.

In this study, we leverage a nationwide population-based cohort and a randomized phase 3 clinical trial to describe the prevalence of patients that would be candidates for adjuvant CDK4/6 inhibitors, their clinicopathologic characteristics and long-term prognosis to assess the efficacy-effectiveness gap between the pivotal clinical trials and the real-world^17^^,^^18^ and to inform clinical practice.

## Methods

### Study design

This is a retrospective analysis of a prospectively collected population-based registry cohort of all patients treated for non-metastatic breast cancer in Sweden. The primary objective of this study is to investigate the prevalence, patient characteristics and long-term prognosis of patients eligible for adjuvant abemaciclib or ribociclib according to the criteria of the monarchE and NATALEE trials, respectively.^19^^,^^20^ The secondary objective was to compare the prognosis of patients treated in real-world and in an international phase 3 trial on dose-dense adjuvant chemotherapy. The population-based study was approved by the ethics review committee in Stockholm (decision 2016/1303-31 August 10th 2016, with amendments 2018/1049-32, 2021-01147 and 2023-02918-02; decision 2019-01908 with amendment 2024-05048-02). The ethical approval granted a waiver for informed consent in this non-interventional collection and analysis of data from registries and patient records. The phase 3 PANTHER trial was approved by ethics review boards at the participating sites and relevant health authorities (initial approval by the Swedish Medicinal Product Agency January 23rd 2007). All patients enrolled in PANTHER provided written informed consent before inclusion and the trial was conducted according to the Declaration of Helsinki.

The reporting of the present study follows the STROBE (Strengthening the Reporting of Observational Studies in Epidemiology) guidelines^21^ and the European Society for Medical Oncology Guidance for Reporting Oncology real-World Evidence (ESMO-GROW) checklist.^22^

### Data source and patient cohorts

All individuals diagnosed with breast cancer between January 1, 2007 and December 31, 2023 in Sweden were included into the study cohort (henceforth named the nationwide cohort). Patients were identified through the National Quality Register for Breast Cancer (in Swedish Nationellt Kvalitetsregister för Bröstcancer, NKBC). NKBC is prospectively maintained and has 99% completeness,^23^ using the Swedish Cancer Register as reference to which all cancer cases are reported by law. The register contains information on patient demographics and clinicopathologic information, including stage according to the Tumor, Node, Metastasis (TNM) classification, receptor expression, grade, Ki67, local treatment (type of breast and axillary surgery, radiotherapy) and (neo)adjuvant systemic therapy, including the date of initiation of adjuvant endocrine therapy. Clinical and pathologic TNM stage were derived and classified according to the American Joint Committee on Cancer 8th edition. For the purposes of this study, patients with invasive breast cancer, ER-positive and HER2-negative, who were treated with endocrine therapy, with or without prior chemotherapy, were identified. Eligible patients were included according to the monarchE and NATALEE criteria (Supplementary Table S1). Patients with in situ breast cancer, missing receptor status or with no information on surgery and endocrine treatment, with metastatic breast cancer (stage IV) or with synchronous bilateral breast cancer diagnosed within three months from primary diagnosis were excluded. Since information on type of disease relapse for the nationwide cohort was limited due to data missingness, overall survival was the endpoint of interest.

For the subset of patients that were treated in the Stockholm–Gotland Region, which comprises approximately 24% of the entire Swedish population, information regarding type of disease relapse (local versus locoregional versus distant, including metastatic site) was available. For this cohort, extensive linkage to several data registers was performed using the unique ten-digit personal identity number assigned to all individuals registered in Sweden. These include the Swedish Prescribed Drug Register, National Cancer Register, the National Patient Register and the Cause of Death Register, providing thus complete information on dispensed drugs, cause of death and comorbidities including other primary cancers.^24^

The second study cohort comprises patients that were enrolled in the phase 3 PANTHER trial. Details on the study design, administered treatment, number and type of relapse and survival rates have been previously presented.^25^, ^26^, ^27^, ^28^, ^29^, ^30^ In short, 2017 patients were enrolled in this academic trial conducted in Sweden, Austria and Germany during 2007–2011. Eligible patients were women aged 18–65 years who had undergone primary surgery for either node-positive, or high-risk node-negative breast cancer (defined as age <35 years, or ER-negative tumor measuring at least 20 mm and grade 3). Patients randomized to experimental treatment received dose dense and tailored dosed adjuvant therapy comprising four cycles of epirubicin and cyclophosphamide (EC) every two weeks, followed by four cycles of docetaxel every two weeks, with chemotherapy dosing adjusted according to a predefined algorithm based on hematologic and non-hematologic toxicities (arm A). Patients randomized to standard treatment received three cycles of FEC (5-fluorouracil and EC) every three weeks, followed by three cycles of docetaxel every three weeks, without dose tailoring (arm B). The same monarchE and NATALEE criteria were applied to identify patients eligible for the current analysis.

### Endpoints

The primary endpoint of this study was OS, defined as time from initiation of adjuvant endocrine therapy to death due to any cause. Distant relapse-free survival (DRFS) as the secondary time-to-failure endpoint was harmonized, based on the updated standardized definitions for efficacy endpoints (STEEP version 2.0^31^), to facilitate comparisons between the PANTHER trial and the Stockholm–Gotland cohort. For DRFS, time was started for PANTHER at randomization, as well as at four months after randomization, serving as the landmark timepoint for initiation of adjuvant endocrine therapy. Patients who died before the landmark time were excluded in the corresponding analysis. For the population-based cohort, DRFS time was started at initiation of adjuvant endocrine therapy. For both cohorts, DRFS time was measured to distant metastasis or death due to any cause, whichever event occurred first. Patients were censored if they were lost to follow-up due to emigration or did not have the event of interest when the study observation period ended (until February 13th, 2024 for the nationwide cohort; January 14th, 2022 for the Stockholm–Gotland region cohort).

### Statistical analysis

In the nationwide cohort analysis, we identified patients with stage I–III ER-positive, HER2-negative breast cancer, who underwent surgery and received adjuvant endocrine therapy. Both numbers and proportions were reported for those who fulfilled both monarchE and NATALEE trials (concordant eligibility), the NATALEE criteria only, or neither of them (non-eligible), respectively. We also calculated annual proportions of newly diagnosed patients with ER-positive, HER2-negative breast cancer meeting the NATALEE or monarchE eligibility criteria, which were illustrated as temporal trends. The baseline clinicopathologic characteristics and individuals meeting each eligibility criterion were separately summarized for each trial-eligible population, with results reported as frequency counts and percentages.

For patients eligible for NATALEE or monarchE, OS was estimated separately using the Kaplan–Meier method and reported as survival rates at 3-, 5-, 7-, and 10-year (or at 9-year for the comparison between PANTHER and the Stockholm–Gotland cohort after 2012, owing to shorter follow-up), along with cumulative incidence curves of all-cause mortality. Considering potential variability in treatments and patient/tumor characteristics over the period from 2007 to 2023, we performed an exploratory analysis comparing event rates between patients diagnosed in 2012–2023 versus those diagnosed in 2007–2011 (when Ki67 was less commonly registered) using Cox proportional hazard models, with proportional hazard assumptions assessed using Schoenfeld residual tests. Adjusted hazard ratios (HR_adj_) with 95% confidence intervals (CI) were presented after taking age, tumor size, nodal status, pathological grade (I, II, and III), chemotherapy use (Yes/No), radiotherapy use (Yes/No), and screening detection (Yes/No) into account, which were chosen based on prior knowledge of clinical relevance to survival outcomes. Similarly, event rates of DRFS between diagnosis years 2012–2020 and 2007–2011 were compared within Stockholm–Gotland region patients. Moreover, sensitivity analyses were conducted excluding patients treated in the earlier three (or five) years of the study period when data completeness was lower, or excluding patients that had less than one (or three) years of follow-up.

For patients with eligibility for the two trials in the nationwide cohort, Kaplan–Meier survival curves were presented, and 10-year OS rates with corresponding 95% CI were estimated for each subgroup. Event rates between subgroups were also compared, with results measured as crude and adjusted HR after controlling for age, grade, chemotherapy use, radiotherapy use, and screening detection using Cox proportional hazard models.

In the second part of this analysis, the prognosis of two independent cohorts of patients with stage I–III ER-positive, HER2-negative BC was compared between the PANTHER trial participants (diagnosed 2007–2011), and a population-based cohort (diagnosed from 2012 to 2020, within the Stockholm–Gotland region), before and after aligning trial inclusion criteria (age under 70 years old and under 65 years old as a sensitivity analysis, pN1-3, and pT1-3). Here, we restricted the multivariable analysis to patients who met the PANTHER trial inclusion criteria, both within the trial and the broader population. This approach supported the positivity assumption necessary for valid regression standardization and enabled a more meaningful interpretation of findings within this specific clinical subgroup, rather than across a heterogeneous population.^32^ For this analysis, the PANTHER trial-based cohort started follow-up at four months after randomization (when 88.9%, 1234/1388 of patients with ER-positive, HER2-negative BC, had initiated adjuvant endocrine therapy).

After alignment, crude OS and DRFS rates were re-estimated accordingly. In multivariable analysis, we first applied regression standardization methods to improve comparability across trial- and population-based cohorts. Specifically, we first fitted a multivariable Cox regression model adjusting for baseline covariates. Using the fitted model, predicted survival probabilities were estimated for all individuals under each exposure category and we then averaged the predicted outcomes over the covariate distribution across the study population. Adjusted cumulative incidence curves and the absolute risk differences at the 5- and 10-year follow-up were estimated based on results from corresponding multivariable Cox regression models,^33^ which included a cohort indicator (representing distinct chemotherapy strategies in combination with adjuvant endocrine therapy), age, pathological grade, tumor size, nodal status, and type of adjuvant endocrine therapy. Then, as a supplementary analysis, exact matching on categorical covariates (grade, nodal status, and type of adjuvant endocrine therapy) and optimal pair matching for continuous ones (age and tumor size) were performed using the matchIt function (version 4.7.0) in R to balance their distributions across two comparison cohorts^34^; following the matched individuals, we applied the log-rank test to examine differences in Kaplan–Meier survival curves.

Missing data for baseline characteristics were reported as numbers and excluded from the calculation of category percentages and complete case analysis. All statistical tests were 2-sided, with p-value <0.05 indicating statistical significance, and statistical analyses were conducted using R (version 4.4.2).

### Role of the funding source

The funding sources had no access or input to any of the following: study design; the collection, analysis, and interpretation of data; the writing of the report; and the decision to submit the paper for publication.

## Results

### Prevalence of eligibility patients for monarchE and NATALEE and baseline characteristics

**Fig. 1:**
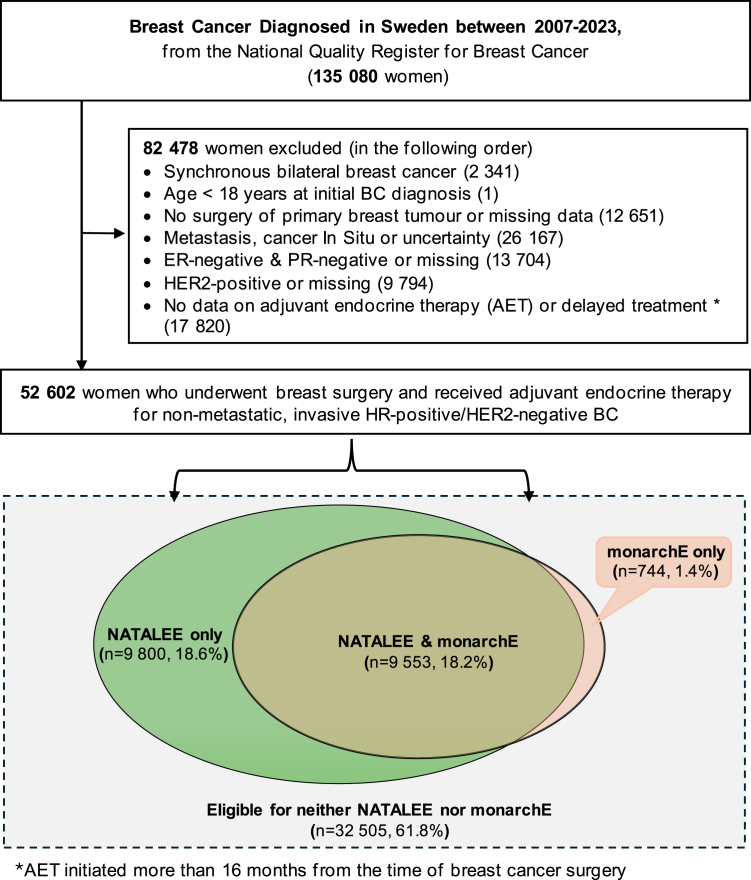
Flowchart of patient selection from the nationwide cohort.

The flowchart describing patient selection from the nationwide cohort is shown in Fig. 1 and Supplementary Figures S1 and S2. Of the 135,080 patients diagnosed with breast cancer in Sweden between 2007 and 2023, a total of 52,602 women underwent surgery for ER-positive, HER2-negative BC and received adjuvant endocrine therapy. Of these, 10,297 patients (19.6%) fulfilled the monarchE criteria, 19,353 patients (36.8%) fulfilled the NATALEE criteria, 9553 patients (18.2%) fulfilled both the monarchE and the NATALEE criteria, 9800 patients (18.6%) fulfilled the NATALEE criteria only and 745 patients (1.4%) fulfilled the monarchE criteria only. The annual proportion of eligible patients for both trials has been stable throughout the study period, with small fluctuations in early years owing mainly to availability of Ki67 (Supplementary Figure S3). The relative proportion of the specific eligibility criteria for monarchE and NATALEE is presented in Supplementary Figure S4. For NATALEE, the most common eligibility criteria were due to anatomical stage IIB (30.1% of all eligible patients) and stage IIA/N1 (25.9% of all eligible patients). The difference in number of eligible patients between the NATALEE and monarchE groups was mainly explained by stage IIA (67.6% versus 16% eligible) and IIB (100% versus 51.2% eligible), as shown in Supplementary Figure S5.

The patients' baseline clinicopathologic characteristics are shown in Table 1. Of note, administration of chemotherapy was less common in the population-based cohort compared to the corresponding clinical trials (monarchE-eligible patients: 68.4% versus 95.4% in the trial; NATALEE-eligible patients: 57.4% versus 88.1% in the trial). In addition, patients with younger age, premenopausal status, and more advanced stage tumors were overrepresented in the two trials compared with the population-based cohort. There was also a temporal trend in the nationwide cohort towards eligibility of patients with older age, less advanced tumors and more frequent use of neoadjuvant therapy in more recent years (Supplementary Table S2).

**Table 1:** Distribution of baseline characteristics for NATALEE- and monarchE-eligible patients.

	NATALEE-eligible	monarchE-eligible	NATALEE trial	monarchE trial
**No. of patients**	n = 19,353 (100%)	n = 10,297 (100%)	n = 5101 (100%)	n = 5637
**Age**				
Median (IQR)	64 (52–73)	63 (51–73)	52 (24–90)	51 (22–89)
<40	694 (3.6)	440 (4.3)		
40–49	2981 (15.4)	1745 (16.9)		
50–64	6281 (32.5)	3342 (32.5)		
65–79	6838 (35.3)	3401 (33.0)		
≥80	2559 (13.2)	1369 (13.3)		
**Menopausal status**				
Premenopausal	4094 (22.7)	2388 (24.9)	2238 (43.9)	2453 (43.5)
Postmenopausal	13,979 (77.3)	7218 (75.1)	2843 (55.7)	3184 (56.5)
Unknown	1280	691		
**Tumor grade**				
Grade 1	1940 (10.1)	702 (6.8)	458 (9.0)	424 (7.5)
Grade 2	11,442 (59.3)	5635 (54.8)	2909 (57.0)	2788 (49.4)
Grade 3	5900 (30.6)	3942 (38.4)	1070 (21.0)	2156 (38.2)
Unknown	71	18	602 (11.8)	266 (4.7)
**pTNM/ypTNM**				
IB	0	744 (7.2)	14 (0.3)	
IIA	9023 (46.6)	2139 (20.8)	1000 (19.6)	676 (12.0)
IIB	5834 (30.1)	2989 (29.0)	1045 (20.5)	776 (13.8)
IIIA	3279 (16.9)	3279 (31.8)	3040 (59.6)	2051 (36.4)
IIIB	141 (0.7)	70 (0.7)		195 (3.4)
IIIC	1076 (5.6)	1076 (10.4)		1912 (34.0)
**Tumor stage**				
T0	6 (0.0)	4 (0.0)		
T1	5919 (30.6)	3797 (37.0)		
T2	11,029 (57.1)	4851 (47.2)		
T3	2212 (11.4)	1529 (14.9)		
T4	162 (0.8)	91 (0.9)		
Missing	25	25		
**pN**				
N0	4749 (24.5)	0	796 (15.6)	14 (0.2)
N1mi	918 (4.7)	1353 (13.1)		2262 (40.1)
N1abc	10,081 (52.1)	5339 (51.9)	2101 (41.2)	
N2	2529 (13.1)	2529 (24.6)	2194 (43.0)	3359 (59.6)
N3	1076 (5.6)	1076 (10.4)		
**Ki67**				
Low (<20)	5367 (34.5)	1625 (18.6)		1926 (34.1)
High (≥20)	10,173 (65.5)	7094 (81.4)		2495 (44.2)
Unknown	3813	1578		1216 (21.6)
**Chemotherapy**	11,108 (57.4)	7043 (68.4)	4494 (88.1)	5376 (95.4)
**Neoadjuvant therapy**	1195 (6.2)	703 (6.8)		
**Type of adjuvant endocrine therapy**				
Aromatase inhibitor (AI)	12,559 (64.9)	6731 (65.4)	5101 (100.0) also includes patients on GRH agonist	3825 (67.8)
Tamoxifen (TAM)	5790 (29.9)	2904 (28.2)		1755 (31.1)
GnRH agonist + AI	387 (2.0)	252 (2.4)		796 (14.1)
GnRH agonist + TAM	551 (2.8)	367 (3.6)		424 (7.5)
Other	66 (0.3)	43 (0.4)		
**Regional radiotherapy**	10,128 (52.3)	7201 (69.9)		

Percentages are calculated on complete data. For comparison, the baseline characteristics in the NATALEE and monarchE trials are presented, as shown in references.^11^^,^^12^

### Prognosis of patients eligible for monarchE and NATALEE

**Fig. 2:**
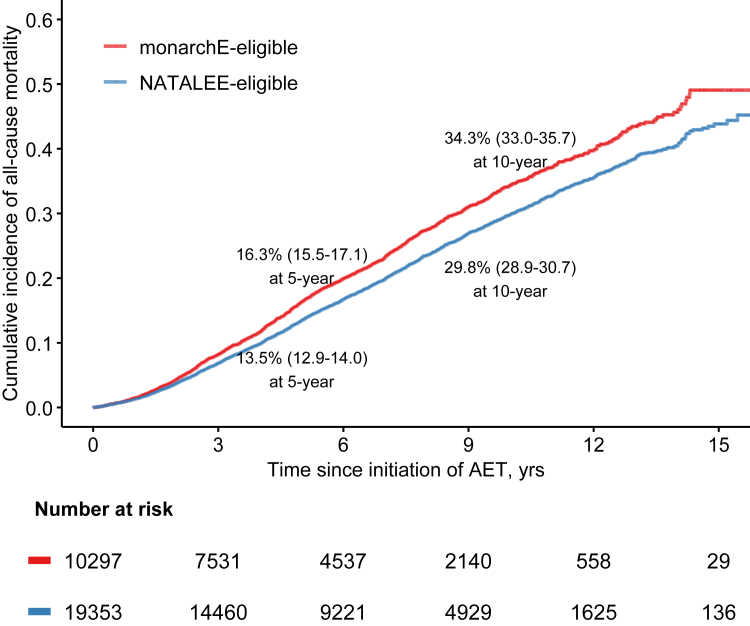
Cumulative incidence curves of all-cause mortality for patients eligible for NATALEE and monarchE in the nationwide cohort. Trial eligibility criteria are presented in Supplementary Table S1. 95% confidence intervals are reported in parentheses.

In the nationwide cohort, median follow-up time was 6.87 years (interquartile range: 3.70–10.18). The 3-year, 5-year and 10-year rates for OS are presented in Table 2. Patients eligible for monarchE generally had worse outcomes compared to those eligible for NATALEE at all time points, with OS reaching 70.2% (95% CI 69.3%–71.1%) for the NATALEE-eligible and 65.7% (95% CI 64.3%–67.0%) for the monarchE-eligible patients at 10 years, for these partially overlapping groups. Cumulative incidence curves of all-cause mortality are presented in Fig. 2. Sensitivity analysis of patients with mature follow-up (diagnosed 2007–2018; median follow-up 9.1 years, interquartile range: 6.8–11.3) revealed similar 10-year OS point estimates (70.2% and 65.5% for the two groups respectively). Additional sensitivity analyses depending on time period of diagnosis and inclusion criteria are presented in Supplementary Table S3.

**Table 2:** Overall survival rates % in subgroups of interest according to trial eligibility in the nationwide cohort.

Patients	No.	3-year rates	5-year rates	7-year rates	10-year rates
NATALEE-eligible	19,353	93.1 (92.8–93.5)	86.5 (86.0–87.1)	80.1 (79.4–80.8)	70.2 (69.3–71.1)
monarchE-eligible	10,297	91.8 (91.2–92.4)	83.7 (82.9–84.5)	76.7 (75.7–77.7)	65.7 (64.3–67.0)
Concordant eligibility	9553	91.3 (90.7–91.9)	82.8 (82.0–83.7)	75.6 (74.5–76.6)	64.3 (62.9–65.7)
NATALEE-only eligible	9800	95.0 (94.5–95.4)	90.1 (89.4–90.8)	84.4 (83.5–85.2)	75.5 (74.4–76.7)
Non-eligible	32,505	97.4 (97.2–97.6)	94.3 (94.0–94.5)	90.4 (90.0–90.8)	84.0 (83.4–84.6)

95% Confidence Intervals are reported in parentheses.

Patient prognosis did not improve statistically significantly during 2012–2023 compared to 2007–2011 (log-rank p = 0.20 for patients eligible for NATALEE; p = 0.067 for monarchE-eligible ones, Supplementary Figure S6) within the current follow-up period. The corresponding HR_adj_ for mortality between NATALEE-eligible patients diagnosed 2012–2023 versus 2007–2011 was 0.94 (95% CI 0.87–1.02), and 0.91 (95% CI 0.82–1.01) for monarchE-eligible patients. In subgroup analysis of the Stockholm–Gotland cohort, the HR_adj_ (i.e., DRFS event rates between individuals diagnosed 2012–2023 and those diagnosed 2007–2011) was 0.94 (95% CI 0.72–1.23) for monarchE-eligible patients. The proportional hazards assumptions for diagnostic period were validated through Schoenfeld residuals tests in the above analyses.

### Prognosis of patients according to trial eligibility

**Fig. 3:**
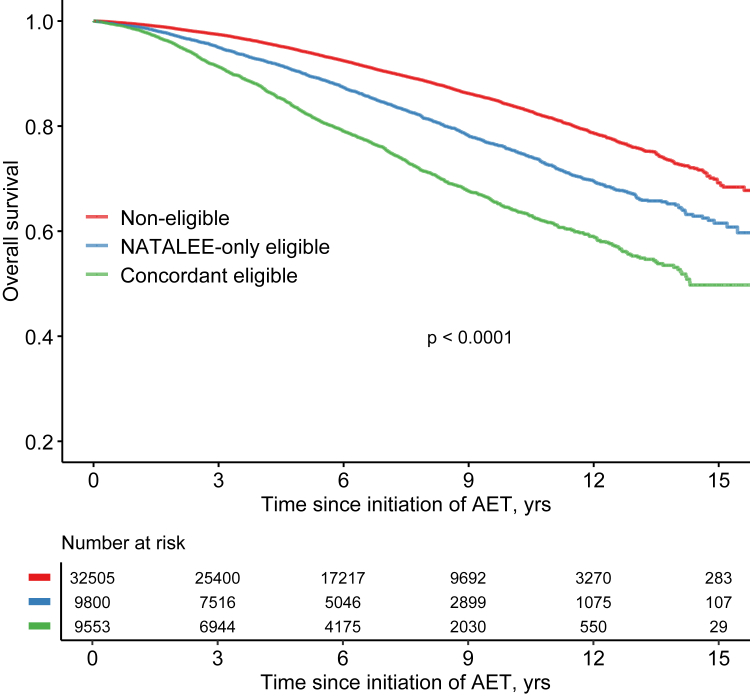
Kaplan–Meier curves of overall survival for patients eligible for both NATALEE and monarchE (concordant group), for NATALEE only or no trials (non-eligible group). Trial eligibility criteria are presented in Supplementary Table S1.

Patients with concordant eligibility for both trials had worse outcomes compared to those eligible for only NATALEE or those not eligible for any trial (Fig. 3). In the nationwide cohort, the 10-year OS rates were 64.3% (95% CI 62.9%–65.7%) for the concordant eligible, 75.5% (95% CI 74.4%–76.7%) for the NATALEE-only eligible and 84.0% (95% CI 83.4%–84.6%) for the non-eligible group (p < 0.0001). The 10-year OS rate of the NATALEE-only eligible patients was worse than non-eligible ones, HR = 1.60 (95% CI 1.51–1.70) and HR_adj_ = 1.41 (95% CI 1.33–1.51), which was interpreted as weighted average over the follow-up period because the proportional hazards assumption was violated. Sensitivity analysis revealed unchanged 10-year OS rates of patients that were diagnosed in 2007–2018 (concordant eligible 64.2%, NATALEE-only eligible 75.7% and non-eligible 83.8%).

The NATALEE-only eligible group was heterogeneous in terms of outcomes, with patients with T4N0-1 (IIIB) disease having considerably worse prognosis than the other stages (Supplementary Figure S7).

### Comparison between a phase 3 trial-based cohort and a population-based cohort

Trial-eligible patients enrolled in the PANTHER trial, compared with those diagnosed 2012–2020 from the Stockholm–Gotland region, had better long-term prognosis. For instance, 9-year DRFS rates for monarchE-eligible patients were 74.5% (95% CI 71.4%–77.7%) versus 65.0% (95% CI 58.6%–72.2%), and for NATALEE-eligible patients 81.0% (95% CI 78.9%–83.3%) versus 68.3% (95% CI 64.7%–72.1%), respectively. Detailed 3-, 5-, 7-, and 9-year OS and DRFS rates for both NATALEE- and monarchE-eligible patients are presented in Supplementary Table S4. Similarly, concordant and discordant eligible patients treated in PANTHER had improved outcomes compared to the population-based cohort.

The above observed difference in prognosis may reflect the more restrictive inclusion criteria applied and inclusion of younger patients in the PANTHER trial-based cohort. In the restricted analysis after alignment, 1380 patients enrolled in PANTHER trial were eligible for NATALEE and 826 patients eligible for monarchE. In the population-based cohort, there were 1345 NATALEE-eligible patients (180 patients had not received chemotherapy) and 645 patients eligible for monarchE (44 patients had not received chemotherapy). In the descriptive analysis of these patients, the differences in the 3-, 5-, 7-, and 9-year OS and DRFS rates between two comparison groups were no longer statistically significant, as indicated by overlapping confidence intervals and the log-rank test p-value (Supplementary Table S5).

We then examined the effect of dose dense chemotherapy for these high-risk patients. Based on the estimated risk of the outcome under two treatment scenarios, if dose-dense versus standard interval treatment was given to the entire pooled study population, the absolute risk decrease for distant metastasis or death at 10 years would be 2.2% (95% CI −2.3% to 6.8%) for NATALEE-eligible and 4.2% (95% CI −2.5% to 10.8%) for monarchE-eligible patients (Supplementary Figure S8).

Finally, 792 matched pairs meeting NATALEE eligibility criteria and 388 matched pairs meeting the monarchE criteria were identified through 1:1 matching. Sensitivity analysis revealed no significant difference in DRFS between matched groups (log-rank p = 0.18 and p = 0.28, respectively; Supplementary Figure S9).

## Discussion

In this study, we describe the nationwide prevalence and outcomes of patients with breast cancer in Sweden who would be candidates for adjuvant abemaciclib or ribociclib according to the corresponding trial criteria. We found that 38.2% of all evaluable patients with ER-positive, HER2-negative breast cancer would be treated with adjuvant CDK4/6 inhibitors, which mirrors other population-based studies,^35^ but not clinical trial-based ones.^15^ The prognosis of these patients is poor, with 10-year OS rates of 65.7% (monarchE criteria) and 70.2% (NATALEE criteria). Notably, 5-year DRFS rates of patients in the population-based cohort were 83.4%, numerically superior to those reported from the control arm of monarchE at 79.2%,^11^ which may hint towards lesser expected absolute benefit from abemaciclib at least at the resource rich setting of the Stockholm–Gotland region. This efficacy-effectiveness gap is further supported by the observed numerical differences in OS between the control arms of the two pivotal trials and the nationwide cohort,^12^ although lack of individual patient data from the two trials, including varying chemotherapy use, precludes in-depth comparisons.

Although monarchE and NATALEE have led to the regulatory approval for adjuvant abemaciclib and ribociclib, respectively, several questions remain unanswered.^36^^,^^37^ An OS benefit has not yet emerged, follow-up is still short considering the long natural history of luminal breast cancer,^1^ early demonstrated benefits in invasive disease-free survival may be prone to informative censoring due to the trials' open label nature,^38^ and the associated financial cost exceeds thresholds of cost-effectiveness in thus far published studies.^39^^,^^40^ Consequently, accurate estimations of the prevalence of the inclusion criteria to the two seminal trials at the population level, and the long-term prognosis of such patients are needed to calculate the overall risk/benefit of introducing adjuvant CDK4/6 inhibitors to the healthcare system.

Our study has several strengths. Firstly, it is a retrospective analysis of a prospectively collected nationwide cohort from a country with an organized mammography screening program which is available to all individuals regardless of employment status or any other socioeconomic or demographic factors, which strengthens the robustness of our results. While previous studies have reported on the prognosis of patients eligible for adjuvant abemaciclib and ribociclib, they suffer from small sample size^15^^,^^16^^,^^35^^,^^41^^,^^42^ and selection bias since most studies concern either patients treated in single/few institutions,^41^ or enrolled in clinical trials with specific inclusion and exclusion criteria.^15^^,^^16^ The latter have reported significantly higher eligibility rates and divergent prognostic implications of the various eligibility subgroups compared with our study, which may also be affected by the organized nationwide screening program for all female residents in Sweden regardless of socioeconomic or other factors. These observations clearly demonstrate the need for complementary population-based studies of sufficient sample size that capture all recent therapeutic advances,^7^ to provide definite answers to pertinent clinical questions. This is further supported by our demonstration of the inherent selection bias in trial-based cohorts as PANTHER compared with population-based ones.

On the other hand, our study suffers from limitations that need to be acknowledged. Firstly, we refer to previously published economic modeling studies as no cost-effectiveness analysis was performed.^39^^,^^40^^,^^43^ Our results, although based on a nationwide cohort that minimizes selection bias, might be specific for Sweden, a country with wide healthcare coverage, organized population-based screening and socioeconomic equity, factors that are associated with improved outcomes. Data missingness concerning Ki67 and gene expression analysis may have limited the study population and impacted the internal validity of our study, especially during the earlier years of the study cohort. Reassuringly, no difference in outcomes during later years was noted among patients eligible for NATALEE, whose inclusion would be impacted by both missing criteria. Data on prior comorbidity were not available in the nationwide and PANTHER trial-base cohorts, therefore could not be adjusted for in the current analyses, which might have introduced residual confounding, while the comparison between the Stockholm–Gotland and PANTHER cohorts concerned patients treated during different time periods to avoid any overlap. Furthermore, Ki67 was assessed using hotspot scoring during the study period, which differs to the method employed in the monarchE trial.^44^ In addition, follow-up may still be short considering the high-risk population and the appearance of the survival curves, with no signs of flattening at the tail-end. Finally, although information on administered adjuvant endocrine treatment and adherence was captured in the Stockholm–Gotland cohort, detailed information for calculating endocrine therapy adherence was not available for the nationwide cohort.

In conclusion, with this nationwide study we describe the prevalence, characteristics and prognosis of patients meeting the eligibility criteria of monarchE and NATALEE. By highlighting the unmet need, specifically the relatively poor survival outcomes in this high-risk population, and estimating the proportion of patients potentially eligible for ribociclib or abemaciclib, healthcare systems can better plan for the implementation and resource allocation of these therapies.

## Contributors

Concept and design: Liu, Binicy, Bergh, Foukakis, Matikas. Acquisition, analysis, or interpretation of data: Liu, Acs, Eriksson Bergman, Valachis, Hartman. Drafting of the initial manuscript: Liu, Binicy, Matikas. Critical revision of the manuscript for important intellectual content: all authors, Statistical analysis: Liu. Obtained funding: Loibl, Gnant, Bergh, Hartman. Administrative, technical, or material support: Acs, Eriksson Bergman, Loibl, Gnant, Untch, Bergh, Hartman, Foukakis, Matikas. Study supervision: Loibl, Gnant, Untch, Valachis, Bergh, Hartman, Foukakis, Matikas. Xingrong Liu and Alexios Matikas had direct access and verified the data supporting the findings of this study. All authors approved the final version of the manuscript and the decision to submit. All authors are part of the academic team behind this study.

## Data sharing statement

The data are not publicly available due to restrictions by Swedish and European law, in order to protect patient privacy. Data are available from the register holder of the Swedish National Breast Cancer Quality Register (NKBC) for researchers with relevant ethical approvals and who meet the criteria for access to confidential data (https://cancercentrum.se/diagnosbehandling/cancerdiagnoser/brost/kvalitetsregister.7359.html). PANTHER trial data are not publicly available due to lack of permission from the Swedish, German and Austrian national ethical review authorities. Data sharing will be reviewed in a case-by-case basis, approved by the steering committee and ethical permit will be applied for. Researchers should contact the corresponding author.

## Declaration of interests

**Sibylle Loibl:** employment as Chief Executive Officer (CEO) at German Breast Group (GBG) Forschungs GmbH; institutional fees for advisory board membership for AbbVie, Amgen, AstraZeneca, Bristol Myers Squibb (BMS), Celgene, DSI, EirGenix, Gilead, GSK, Lilly, Merck, Novartis, Olema, Pfizer, Pierre Fabre, Relay Therapeutics, Roche, Sanofi and Seagen; institutional fees as an invited speaker for AstraZeneca, DSI, Gilead, Medscape, Novartis, Pfizer, Roche, Seagen and Stemline-Menarini; institutional research grants from AbbVie, AstraZeneca, BMS/Celgene, Daiichi Sankyo, Immunomedics/Gilead, Molecular Health, Stemline-Menarini, Novartis, Pfizer and Roche; institutional funding from Greenwich Life Sciences; institutional licensing fees from VMscope GmbH; a role as a steering committee member (non-financial interest) for AstraZeneca, Daiichi Sankyo, Immunomedics/Gilead, Novartis, Pfizer, Roche and Seagen; a role as a Principal Investigator (PI) for Pfizer, AstraZeneca (non-financial interest). **Michael Gnant:** personal fees for advisory board membership for Eli Lilly, MSD, Novartis, Bayer and Menarini-Stemline; personal fees as an invited speaker for AstraZeneca, Daiichi Sankyo, Eli Lilly, EPQ Health, MSD, Novartis and Pierre Fabre; personal fees for an expert testimony for Veracyte; membership of the Board of Directors at Austrian Breast and Colorectal Cancer Study Group (ABCSG) GmbH and ABCSG Research Services GmbH; a role as a steering committee member for AstraZeneca (non-financial interest) and Eli Lilly (non-financial interest); a role as trial Chair for Pfizer (non-financial interest). **Michael Untch:** personal fees for lectures and/or consultancy from Agendia, AstraZeneca, Daiichi Sankyo, Eisai Gilead, Lilly Deutschland, MSD, Myriad Genetics, Novartis, Pierre Fabre, Pfizer, Roche, Sanofi Aventis, Seagen, Stemline. **Antonios Valachis:** unrestricted research funding paid to institution by Roche and MSD. **Jonas Bergh:** research funding to institution from Amgen, AstraZeneca, Bayer, Merck, Pfizer, Roche and Sanofi; honoraria from UpToDate paid to Asklepios Cancer Research AB; head of advisory board at Stratipath AB; Coronis and Asklepios Cancer Research AB hold shares of Stratipath AB; honoraria for lectures/educational conferences for postgraduates courses from AstraZeneca paid to Coronis and Asklepios Cancer Research AB. **Johan Hartman:** Leadership and ownership interests in Stratipath AB. Speaker: Gilead. Honoraria from MSD, Pfizer, Lilly. Research funding (paid to institution): Novartis, Cepheid. **Theodoros Foukakis:** invited Speaker, Payment to Institution: Roche, Astra Zeneca, Gilead Sciences, Novartis, Daiichi Sankyo; royalties, personal: Wolters Kluwer (authorship of two chapters in UpToDate); clinical trial support to Institution: Astra Zeneca, Coordinating PI (research grant and study drug), Novartis, Coordinating PI (research grant and study drug), Veracyte, Coordinating PI (discount on the ProsignaPAM50 assay in ARIADNE clinical trial). **Alexios Matikas:** speaker (no personal fees): Roche, Seagen; consultant (no personal or institutional fees): Roche, AstraZeneca, Veracyte; research funding paid to institution by Merck, AstraZeneca, Novartis, Veracyte; advisory board: Nordic Pharma (no personal or institutional fees).

All the other authors had no potential conflicts of interest to disclose.

## Appendix A Supplementary data

Supplementary Tables and Figures

Statistical Analysis Plan
