# Supplementary material for: Prevalence and prognosis of patients with breast cancer eligible for adjuvant abemaciclib or ribociclib: a nationwide population-based study
Source: Lancet Reg Health Eur. 2025 Sep 29;59:101471. doi: 10.1016/j.lanepe.2025.101471 (PMC12513154; doi:10.1016/j.lanepe.2025.101471)

Supplementary material to

Prevalence and prognosis of patients with breast cancer eligible for adjuvant abemaciclib or ribociclib: a nationwide population-based study

Xingrong Liu, Behnaz Binicy, Balazs Acs, Louise Eriksson Bergman, Sibylle Loibl, Michael Gnant, Michael Untch, Antonios Valachis, Jonas Bergh, Johan Hartman, Theodoros Foukakis, Alexios Matikas

Table of Contents

Table S1. Eligibility criteria of NATALEE and monarchE 2
Table S2. Patient characteristics according to trial eligibility and time period 3
Table S3. Sensitivity analyses 5
Table S4. Survival rates in PANTHER and Stockholm cohorts before alignment 6
Table S5. Survival rates in PANTHER and Stockholm cohorts after alignment 7
Figure S1. Patient flow according to NATALEE eligibility 8
Figure S2. Patient flow according to monarchE eligibility 9
Figure S3. Trial eligibility per calendar year in the nationwide cohort 10
Figure S4. Specific trial eligibility criteria in the nationwide cohort 11
Figure S5. Trial eligibility by TNM stage in the nationwide cohort 12
Figure S6. Kaplan-Meier curves for OS according to trial eligibility and time period 13
Figure S7. Kaplan-Meier curves for OS of trial-eligible patients by TNM stage 14
Figure S8. Adjusted cumulative incidence curves for DRFS 15
Figure S9. Kaplan-Meier curves for DRFS of trial-eligible matched patients 16

**Supplementary Table 1.** Eligibility criteria for the NATALEE and monarchE trials			
**AJCC Stage**	**TNM**	**NATALEE**	**monarchE**
I	T1N0	No	No
IIA	T0N1	Yes	If grade 3 or Ki67 ≥20%
	T1N1	Yes	If grade 3 or Ki67 ≥20%
	T2N0	If grade 3 or high genomic risk or Ki67 ≥20%	No
IIB	T2N1	Yes	If grade 3 or Ki67 ≥20%
	T3N0	Yes	No
IIIA	T0N2	Yes	Yes
	T1N2	Yes	Yes
	T2N2	Yes	Yes
	T3N1	Yes	Yes
	T3N2	Yes	Yes
IIIB	T4N0	Yes	No
	T4N1	Yes	If grade 3 or Ki67 ≥20% or size ≥5 cm
	T4N2	Yes	Yes
IIIC	T1-4N3	Yes	Yes

Supplementary Table 2. Distribution of baseline characteristics for NATALEE- and monarchE-eligible patients, stratified by diagnosis time period. Percentages are calculated on complete data.

	NATALEE-eligible, n (%)		monarchE-eligible, n (%)	
	**2007-2012**	**2013-2023**	**2007-2012**	**2013-2023**
**No. of patients**	4337 (22.4)	15016 (77.6)	1849 (18.0)	8448 (82.0)
**Age**				
Median (IQR)	63 (52-72)	64 (53-74)	62 (51-72)	63 (51-73)
< 40	152 (3.5)	542 (3.6)	84 (4.5)	356 (4.2)
40-49	685 (15.8)	2296 (15.3)	311 (16.8)	1434 (17.0)
50-64	1601 (36.9)	4680 (31.2)	663 (35.9)	2679 (31.7)
65-79	1443 (33.3)	5395 (35.9)	581 (31.4)	2820 (33.4)
≥ 80	456 (10.5)	2103 (14.0)	210 (11.4)	1159 (13.7)
**Menopausal status**				
Premenopausal	916 (23.4)	3178 (22.4)	417 (25.0)	1971 (24.8)
Postmenopausal	2998 (76.6)	10981 (77.6)	1252 (75.0)	5966 (75.2)
Unknown	423	857	180	511
**Tumor grade**				
Grade 1	506 (11.7)	1434 (9.6)	84 (4.6)	618 (7.3)
Grade 2	2324 (53.7)	9118 (61.0)	771 (41.8)	4864 (57.7)
Grade 3	1499 (34.6)	4401 (29.4)	991 (53.7)	2951 (35.0)
Unknown	8	63	3	15
**pTNM/ypTNM**				
IB	0	0	21 (1.1)	723 (8.6)
IIA	1864 (43.0)	7159 (47.7)	277 (15.0)	1862 (22.0)
IIB	1342 (30.9)	4492 (29.9)	431 (23.3)	2558 (30.3)
IIIA	813 (18.7)	2466 (16.4)	813 (44.0)	2466 (29.2)
IIIB	22 (0.5)	119 (0.8)	11 (0.6)	59 (0.7)
IIIC	296 (6.8)	780 (5.2)	296 (16.0)	780 (9.2)
**Tumor stage**				
T0	0 (0.0)	6 (0.0)	0 (0.0)	4 (0.0)
T1	1554 (35.8)	4365 (29.1)	541 (29.3)	3256 (38.6)
T2	2273 (52.4)	8756 (58.4)	961 (52.0)	3890 (46.2)
T3	481 (11.1)	1731 (11.5)	329 (17.8)	1200 (14.2)
T4	27 (0.6)	135 (0.9)	16 (0.9)	75 (0.9)
Missing	2	23	2	23
**pN**				
N0	712 (16.4)	4037 (26.9)	0	0
N1mi	67 (1.5)	851 (5.7)	43 (2.3)	1310 (15.5)
N1abc	2598 (59.9)	7483 (49.8)	846 (45.8)	4493 (53.2)
N2	664 (15.3)	1865 (12.4)	664 (35.9)	1865 (22.1)
N3	296 (6.8)	780 (5.2)	296 (16.0)	780 (9.2)
**Ki67**				
Low (<20)	196 (35.7)	5171 (34.5)	59 (20.5)	1566 (18.6)
High (≥20)	352 (64.2)	9821 (65.5)	229 (79.5)	6865 (81.4)
Unknown	3789	24	1561	17
**Chemotherapy**	2418 (55.8)	8690 (57.9)	1288 (69.8)	5755 (68.1)

**Neoadjuvant therapy**	25 (0.6)	1170 (7.8)	13 (0.7)	690 (8.2)

**Supplementary Table 3.** Proportions of incident cases, category distribution by trial inclusion criteria, and survival rates at 3, 5, 7, and 10 years among patients diagnosed in 2010-2022, 2012-2020, or 2007-2023, stratified by NATALEE- and monarchE-eligibility			
**NATALEE-eligible**			
**Diagnostic period**	**2007-2023**	**2010-2022**	**2012-2020**
**Incident cases (proportion)**	19353 (36.8%)	17942 (36.8%)	13008 (36.6%)
**Category by trial inclusion criteria, n (%)**			
Stage III	4496 (23.2)	4143 (23.1)	2929 (22.5)
Stage IIB	5834 (30.1)	5374 (30.0)	3831 (29.5)
Stage IIA (N1)	5007 (25.9)	4575 (25.5)	3296 (25.3)
Stage IIA (N0 & Grade3)	2062 (10.7)	1932 (10.8)	1422 (10.9)
Stage IIA (N0 & Grade2 & Ki67 ≥ 20%)	1954 (10.1)	1918 (10.7)	1530 (11.8)
**Overall survival (95% CIs), %**			
3-year	93.1 (92.8-93.5)	93.2 (92.8-93.6)	93.3 (92.9-93.8)
5-year	86.5 (86.0-87.1)	86.7 (86.1-87.2)	86.9 (86.3-87.5)
7-year	80.1 (79.4-80.8)	80.3 (79.6-81.0)	80.6 (79.8-81.4)
10-year	70.2 (69.3-71.1)	70.5 (69.5-71.4)	70.5 (69.4-71.7)
**monarchE-eligible**			
**Diagnostic period**	**2007-2023**	**2010-2022**	**2012-2020**
**Incident cases (proportion)**	10297 (19.6%)	9699 (19.9%)	7261 (20.4%)
**Category by trial inclusion criteria, n (%)**			
≥ 4 positive axillary lymph nodes (ALNs)	3608 (35.0)	3323 (34.3)	2362 (32.5)
1-3 positive ALNs & (tumour sizee≥5cm or grade 3)	3543 (34.4)	3292 (33.9)	2375 (32.7)
1-3 positive ALNs & tumour size<5cm & grade 1-2 & Ki-67≥20%	3146 (30.6)	3084 (31.8)	2524 (34.8)
**Overall survival (95% CIs), %**			
3-year	91.8 (91.2-92.4)	91.9 (91.3-92.5)	92.3 (91.6-92.9)
5-year	83.7 (82.9-84.5)	84.0 (83.2-84.9)	84.6 (83.7-85.5)
7-year	76.7 (75.7-77.7)	77.1 (76.1-78.2)	78.2 (77.1-79.3)
10-year	65.7 (64.3-67.0)	66.4 (65.0-67.8)	67.6 (66.0-69.2)

**Supplementary Table 4.** Crude survival rates *before* alignment on key inclusion criteria						
**Overall survival rates % (95% CI)**						
**Patients**	**Groups**	**No.**	**3-year rates**	**5-year rates**	**7-year rates**	**9-year rates**
NATALEE-eligible	Stockholm	2807	94.8 (94.0–95.7)	89.1 (87.7–90.5)	82.6 (80.6-84.6)	74.2 (70.9–77.6)
	PANTHER	1384	97.8 (97.0–98.6)	93.6 (92.3–94.9)	90.1 (88.5-91.8)	87.9 (86.1–89.8)
monarchE-eligible	Stockholm	1502	93.9 (92.7–95.2)	86.8 (84.8–89.0)	81.5 (78.8-84.4)	71.8 (66.1–78.0)
	PANTHER	830	96.8 (95.6–98.0)	91.2 (89.3–93.2)	86.5 (84.1-89.0)	84.2 (81.6–86.9)
Concordant eligibility	Stockholm	1357	93.5 (92.2–95.0)	85.7 (83.4–88.0)	80.0 (77.1-83.1)	70.4 (64.6–76.7)
	PANTHER	830	96.8 (95.6–98.0)	91.2 (89.3–93.2)	86.5 (84.1-89.0)	84.2 (81.6–86.9)
NATALEE-only eligible	Stockholm	1450	96.1 (95.0-97.1)	92.0 (90.4-93.7)	84.9 (82.4-87.5)	77.0 (73.0-81.2)
	PANTHER	554	99.3 (98.6–100)	97.2 (95.8–98.6)	95.5 (93.7-97.3)	93.6 (91.4–95.8)
Non-eligible	Stockholm	5756	97.6 (97.2–98.0)	95.2 (94.6–95.9)	91.7 (90.7-92.6)	88.7 (87.4–90.1)
(or missing data)	PANTHER	4	*	*		*
**Distant relapse-free survival rates % (95% CI)**						
**Patients**	**Groups**	**No.**	**3-year rates**	**5-year rates**	**7-year rates**	**9-year rates**
NATALEE-eligible	Stockholm	2807	91.3 (90.2-92.4)	85.7 (84.2-87.2)	78.6 (76.5-80.7)	68.3 (64.7-72.1)
	PANTHER	1384	94.2 (92.9-95.4)	89.2 (87.5-90.9)	85.1 (83.2-87.1)	81.0 (78.9-83.3)
monarchE-eligible	Stockholm	1502	89.7 (88.1-91.4)	83.4 (81.2-85.6)	77.1 (74.2-80.1)	65.0 (58.6-72.2)
	PANTHER	830	91.8 (89.9-93.7)	85.4 (83.0-87.8)	79.8 (77.0-82.7)	74.5 (71.4-77.7)
Concordant eligibility	Stockholm	1357	89.1 (87.3-90.8)	82.1 (79.8-84.6)	75.4 (72.3-78.6)	63.1 (56.6-70.4)
	PANTHER	830	91.8 (89.9-93.7)	85.4 (83.0-87.8)	79.8 (77.0-82.7)	74.5 (71.4-77.7)
NATALEE-only eligible	Stockholm	1450	93.4 (92.0-94.8)	89.0 (87.1-90.8)	81.4 (78.8-84.2)	72.0 (67.6-76.6)
	PANTHER	554	97.8 (96.5-99.0)	94.9 (93.1-96.8)	93.0 (90.8-95.2)	90.9 (88.3-93.5)
Non-eligible	Stockholm	5756	96.8 (96.3-97.2)	93.7 (93.0-94.4)	89.9 (88.9-90.9)	86.3 (84.8-87.9)
(or missing data)	PANTHER	4	*	*		*

**Supplementary Table 5.** Crude survival rates *after* alignment on key inclusion criteria							
**Overall survival rates % (95% CI)**							
**Patients**	**Groups**	**No.**	**3-year rates**	**5-year rates**	**7-year rates**	**9-year rates**	**p**
NATALEE-eligible	Stockholm	1345	98.0 (97.2-98.8)	94.0 (92.5-95.6)	90.4 (88.2-92.6)	88.1 (85.3-90.9)	0.59
	PANTHER	1380	97.5 (96.6-98.3)	93.2 (91.8-94.5)	89.8 (88.1-91.5)	87.3 (85.4-89.2)	
monarchE-eligible	Stockholm	645	96.9 (95.5-98.4)	91.1 (88.4-93.8)	87.1 (83.7-90.8)	82.8 (78.3-87.6)	0.98
	PANTHER	826	96.3 (95.0-97.6)	90.8 (88.8-92.8)	86.1 (83.7-88.6)	83.4 (80.7-86.1)	
**Distant relapse-free survival rates % (95% CI)**							
**Patients**	**Groups**	**No.**	**3-year rates**	**5-year rates**	**7-year rates**	**9-year rates**	**p**
NATALEE-eligible	Stockholm	1345	94.3 (93.0-95.7)	91.2 (89.5-93.0)	86.1 (83.6-88.6)	80.7 (76.2-85.4)	0.31
	PANTHER	1380	93.2 (91.9-94.6)	88.8 (87.1-90.5)	84.3 (82.3-86.3)	80.5 (78.3-82.8)	
monarchE-eligible	Stockholm	645	91.2 (88.9-93.6)	86.5 (83.5-89.6)	80.4 (76.4-84.5)	74.3 (67.8-81.5)	0.75
	PANTHER	826	90.7 (88.7-92.7)	84.7 (82.3-87.3)	79.1 (76.2-82.0)	73.8 (70.7-77.1)	

Supplementary Figure 1. Patient flow according to specific eligibility criteria for NATALEE

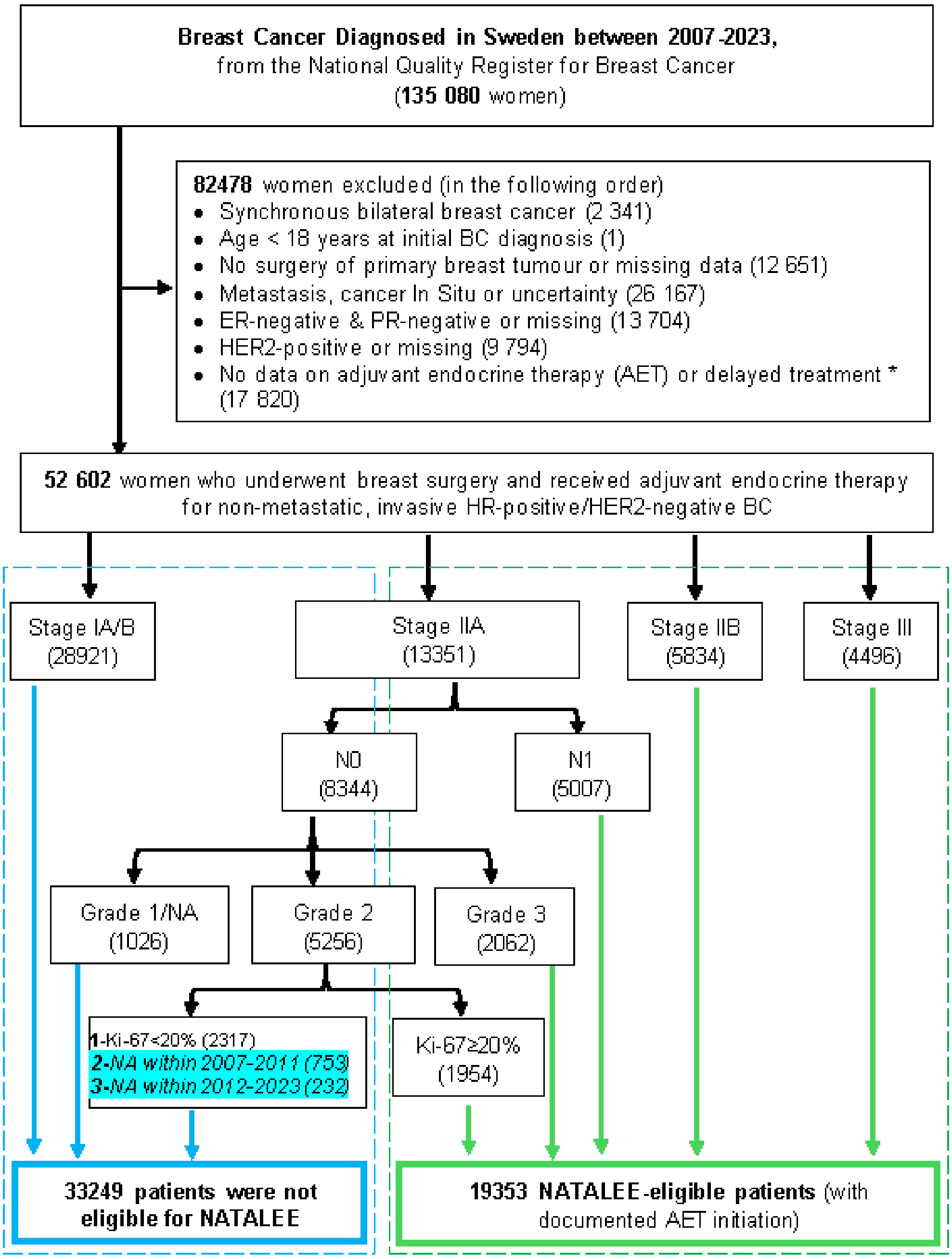

* AET initiated more than 16 months from the time of breast cancer surgery. NA, not available.

Supplementary Figure 2. Patient flow according to specific eligibility criteria for monarchE

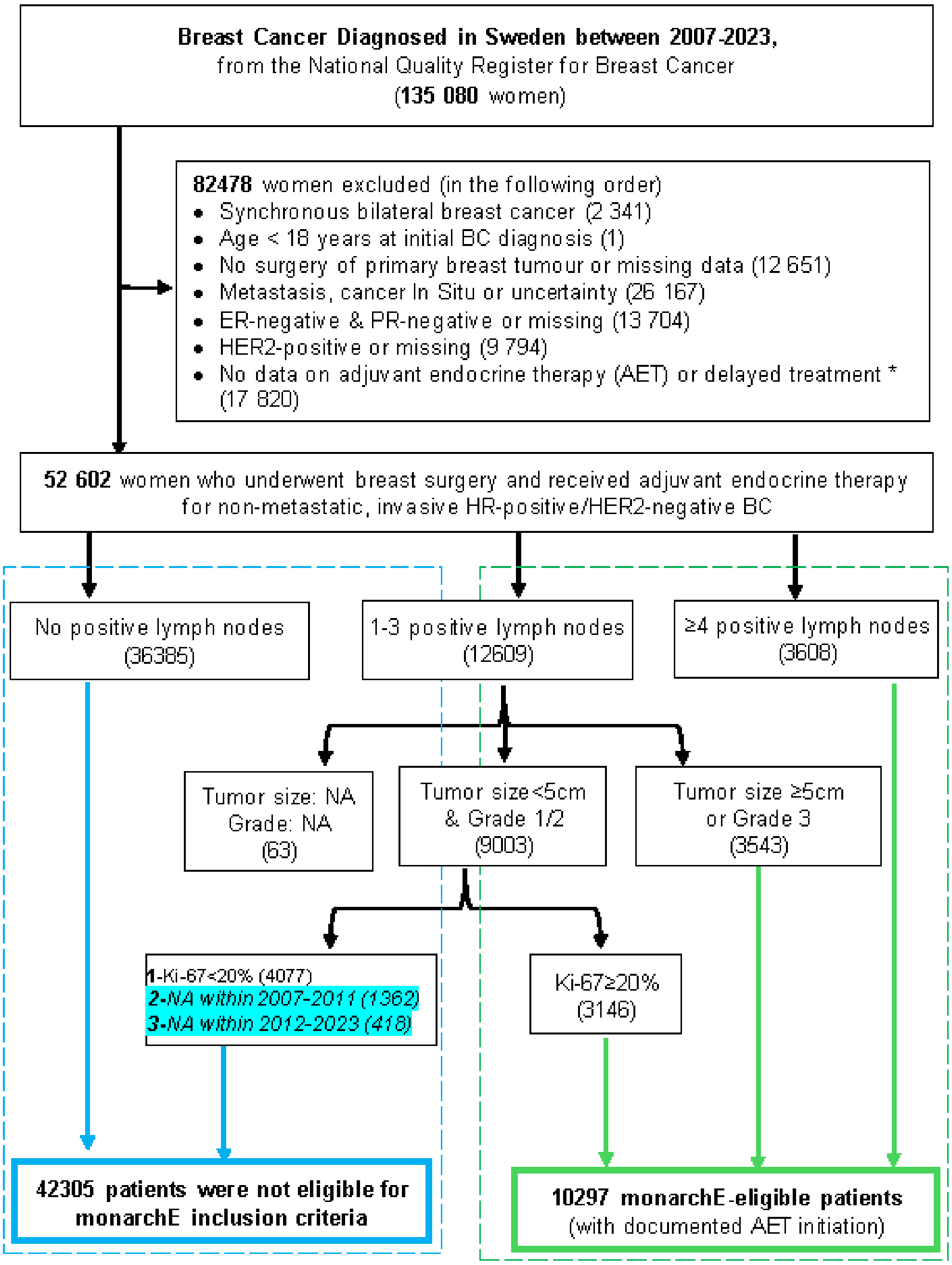

* AET initiated more than 16 months from the time of breast cancer surgery. NA, not available.

Supplementary Figure 3. Proportion of NATALEE- and monarchE-eligible patients in the nationwide cohort per calendar year

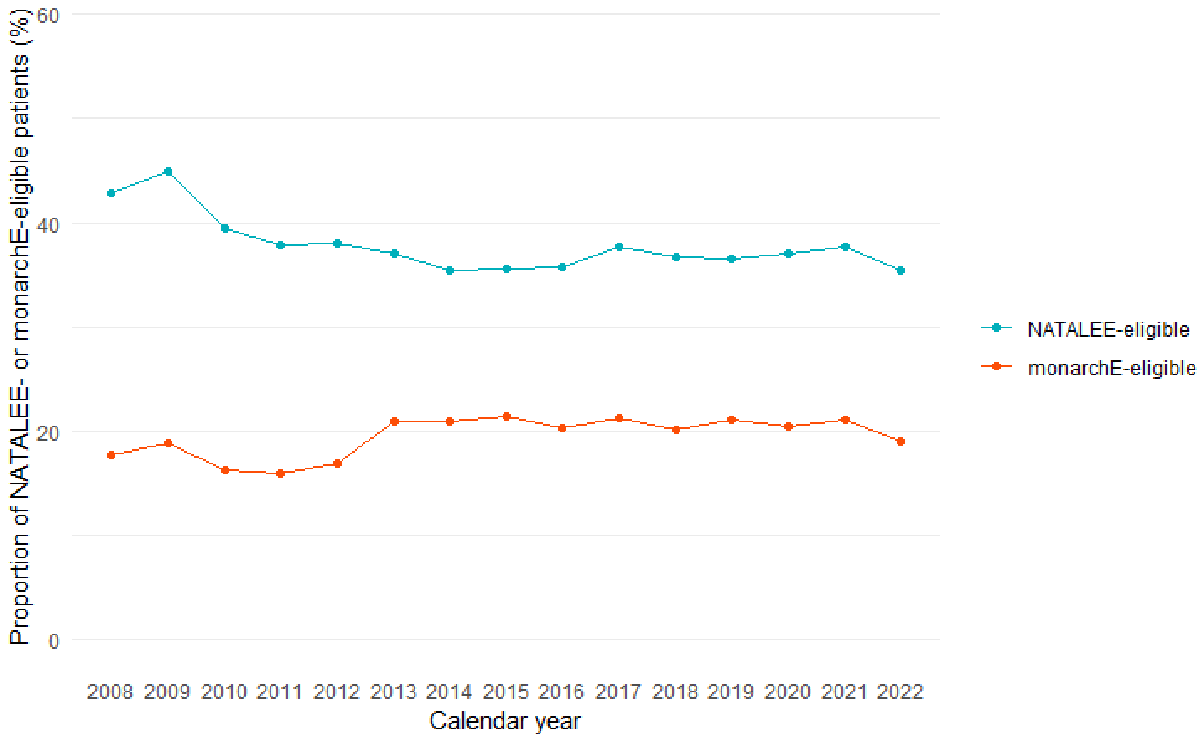

Supplementary Figure 4. Specific eligibility criteria for NATALEE and monarchE in the nationwide cohort

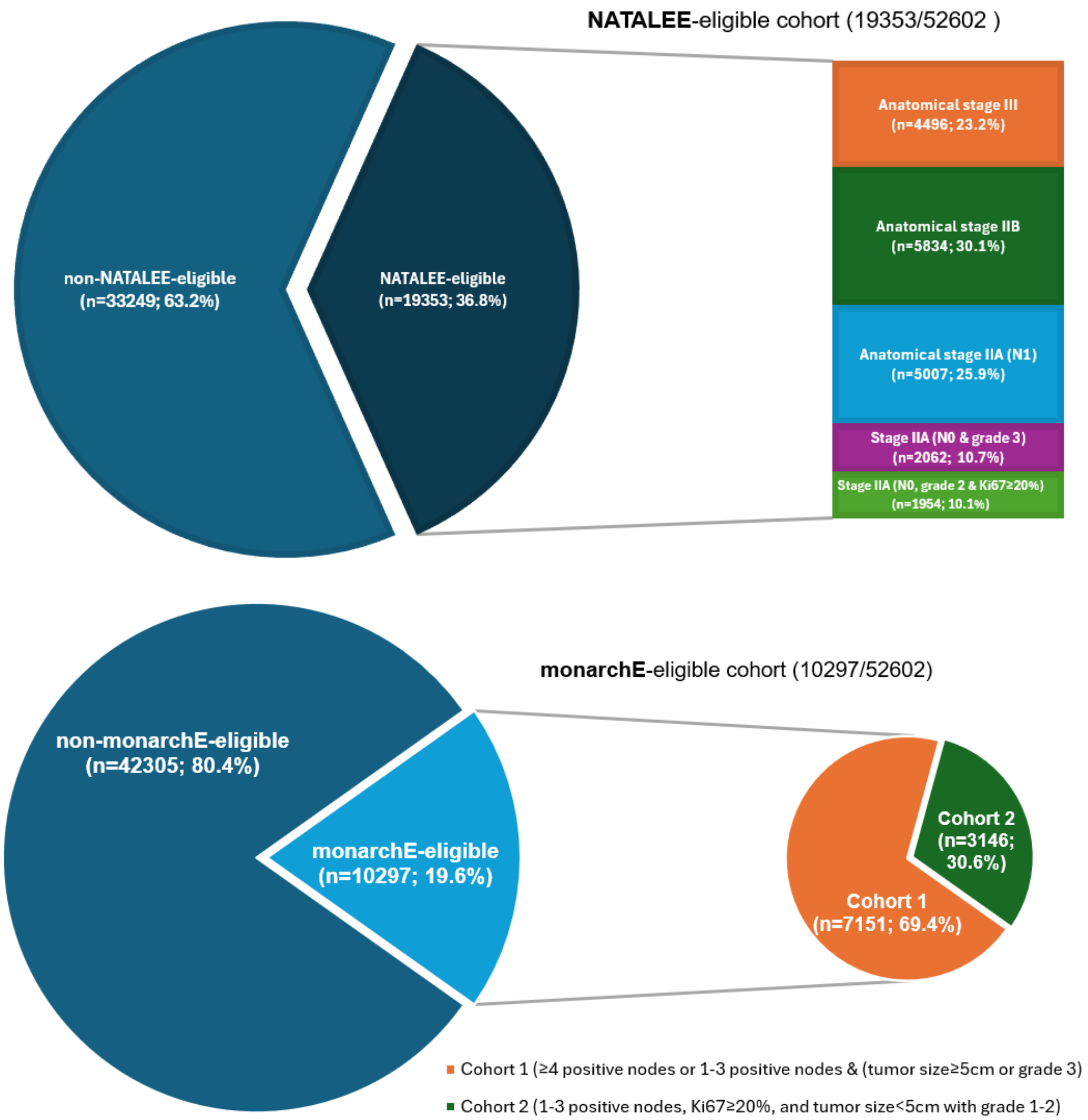

Supplementary Figure 5. Distribution of NATALEE- and monarchE-eligible patients in the nationwide cohort by TNM stage

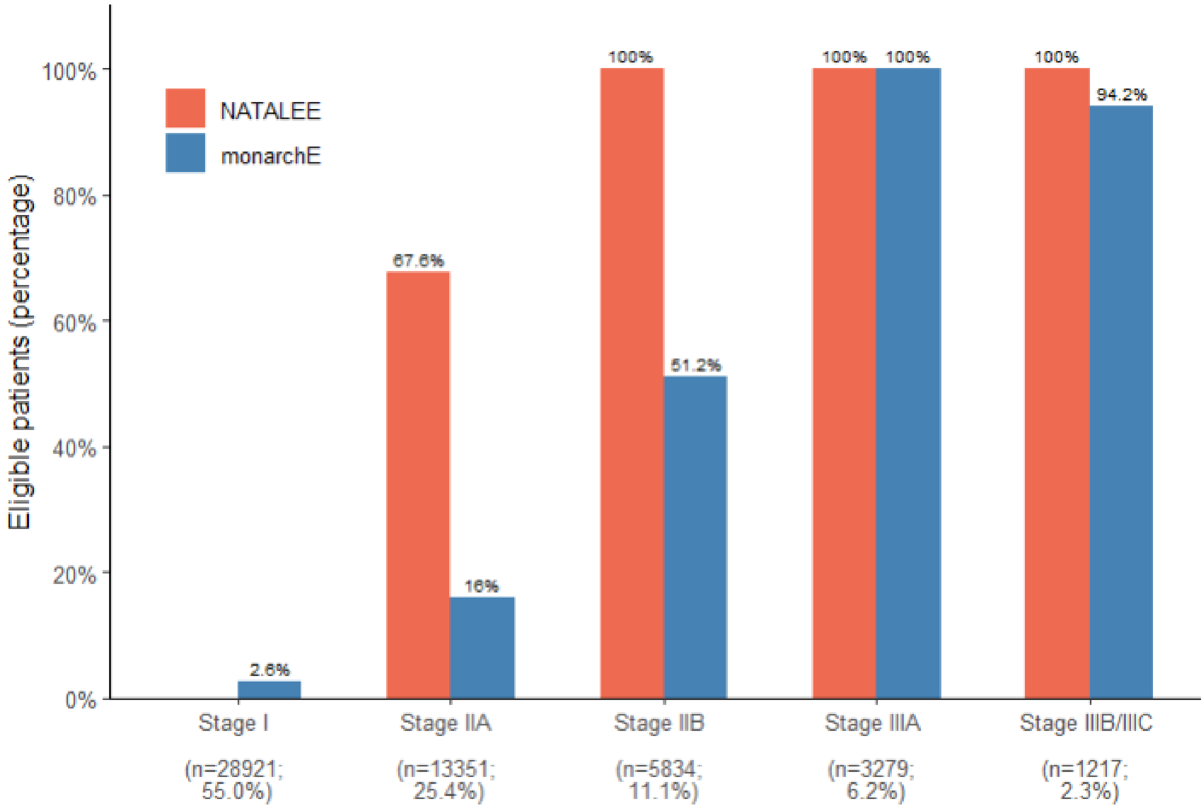

Supplementary Figure 6. Kaplan-Meier curves for overall survival of patients eligible for NATALEE (A) and monarchE (B) according to time period

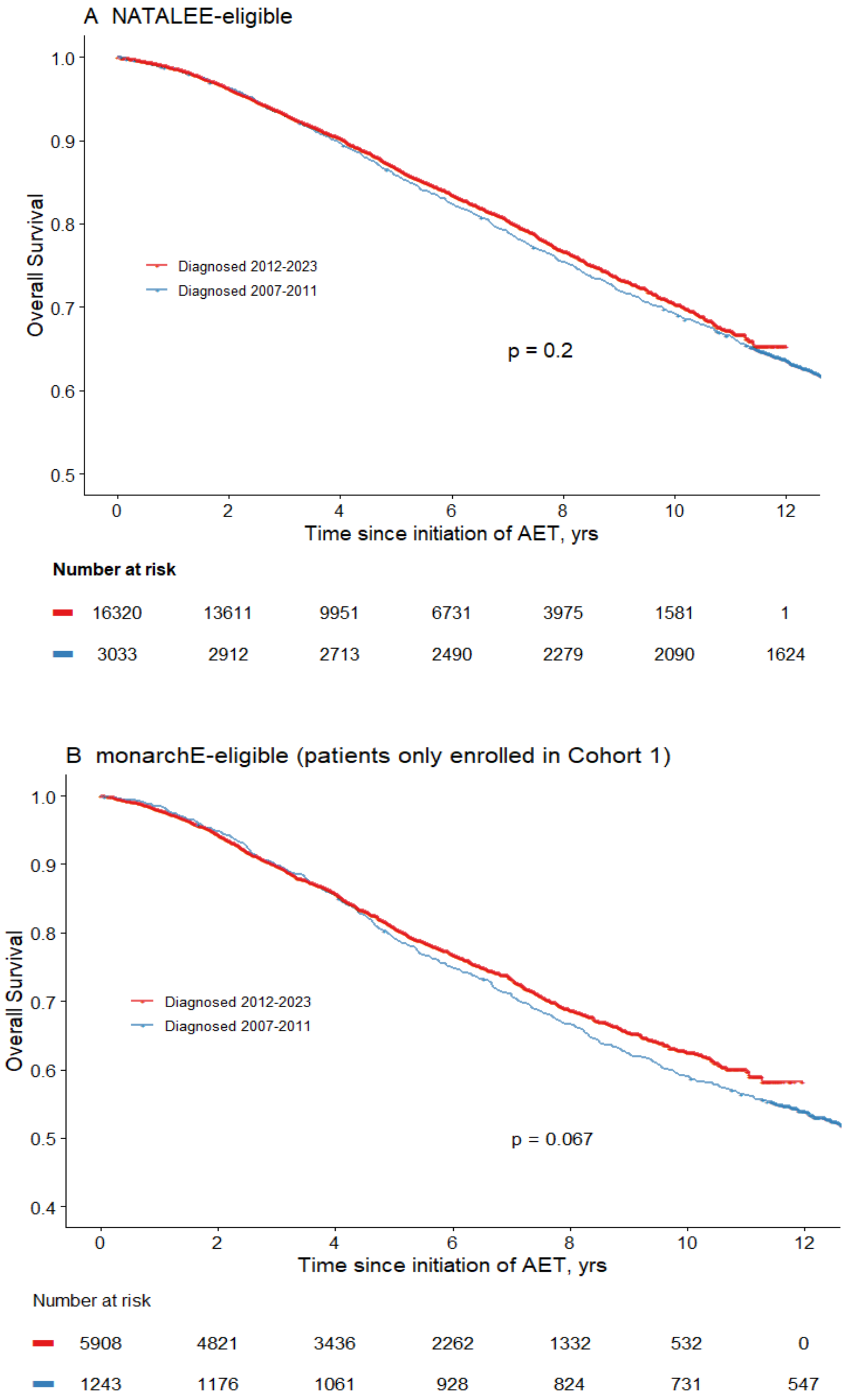

Supplementary Figure 7. Kaplan-Meier curves for overall survival of discordant eligible patients in the nationwide cohort by TNM stage

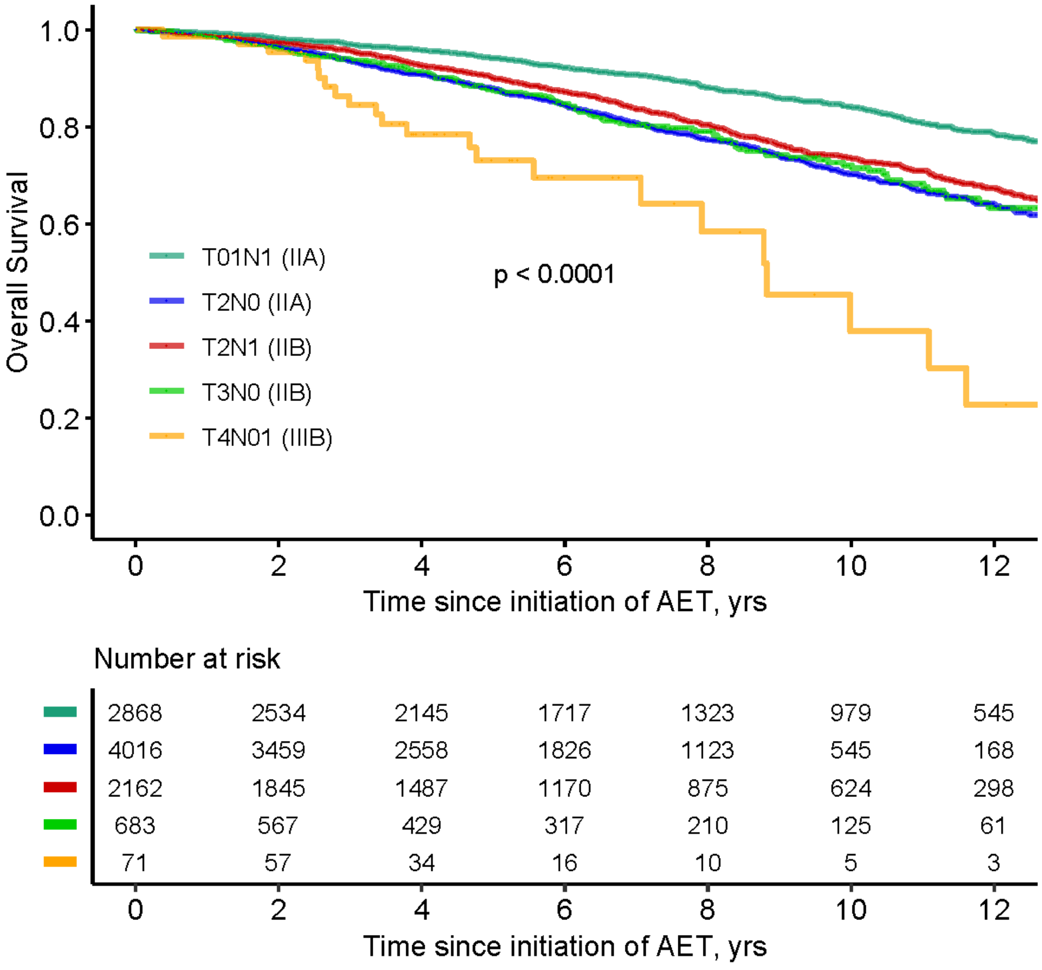

Supplementary Figure 8. Adjusted cumulative incidence curves of distant relapse or death following regression standardization for patients eligible for NATALEE (A) and monarchE (B). PANTHER arm A: dose dense chemotherapy. PANTHER arm B: standard interval chemotherapy

A NATALEE-eligible

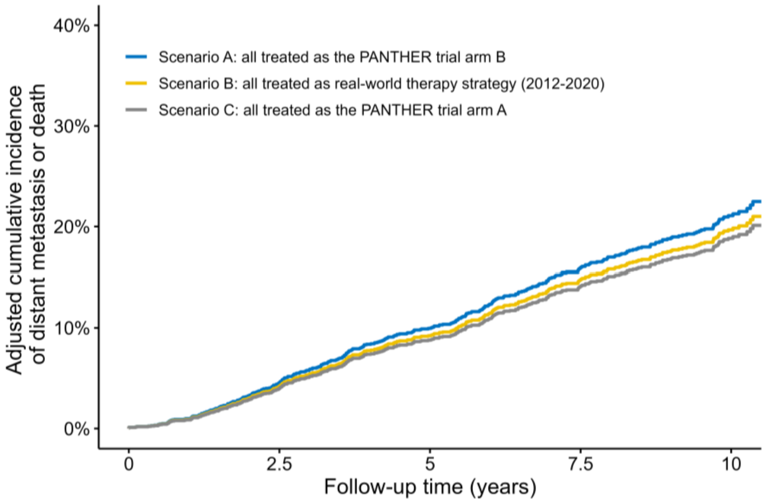

	5 y	10 y
Adjusted cumulative incidence at 5 and 10 y (95% CI), %		
Scenario A	10.0 (8.0 to 12.0)	21.3 (18.0 to 24.5)
Scenario B	9.3 (7.6 to 11.0)	19.8 (16.3 to 23.3)
Scenario C	8.8 (7.0 to 10.7)	19.0 (15.7 to 22.3)
Risk difference at 5 and 10 y (95% CI), %		
Scenario A to Scenario C	1.2 (-1.5 to 3.8)	2.2 (-2.3 to 6.8)
Scenario B to Scenario C	0.4 (-2.1 to 2.9)	0.8 (-3.9 to 5.6)
Scenario A to Scenario B	0.7 (-1.9 to 3.3)	1.4 (-3.4 to 6.2)

B monarchE-eligible

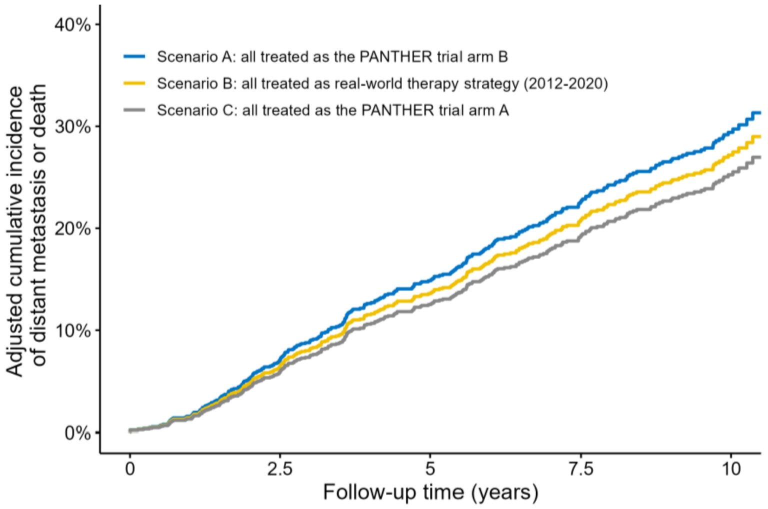

	5 y	10 y
Adjusted cumulative incidence at 5 and 10 y (95% CI), %		
Scenario A	15.0 (11.9 to 18.0)	29.7 (25.0 to 34.5)
Scenario B	13.7 (10.7 to 16.6)	27.5 (22.0 to 32.9)
Scenario C	12.6 (9.7 to 15.5)	25.5 (20.7 to 30.3)
Risk difference at 5 and 10 y (95% CI), %		
Scenario A to Scenario C	2.3 (-1.8 to 6.5)	4.2 (-2.5 to 10.8)
Scenario B to Scenario C	1.1 (-3.0 to 5.2)	1.9 (-5.2 to 9.1)
Scenario A to Scenario B	1.3 (-3.0 to 5.5)	2.2 (-4.9 to 9.4)

Supplementary Figure 9. Kaplan-Meier curves for distant relapse-free survival of matched NATALEE-eligible (A) and monarchE-eligible (B) patients in PANTHER and in the Stockholm population-based cohort

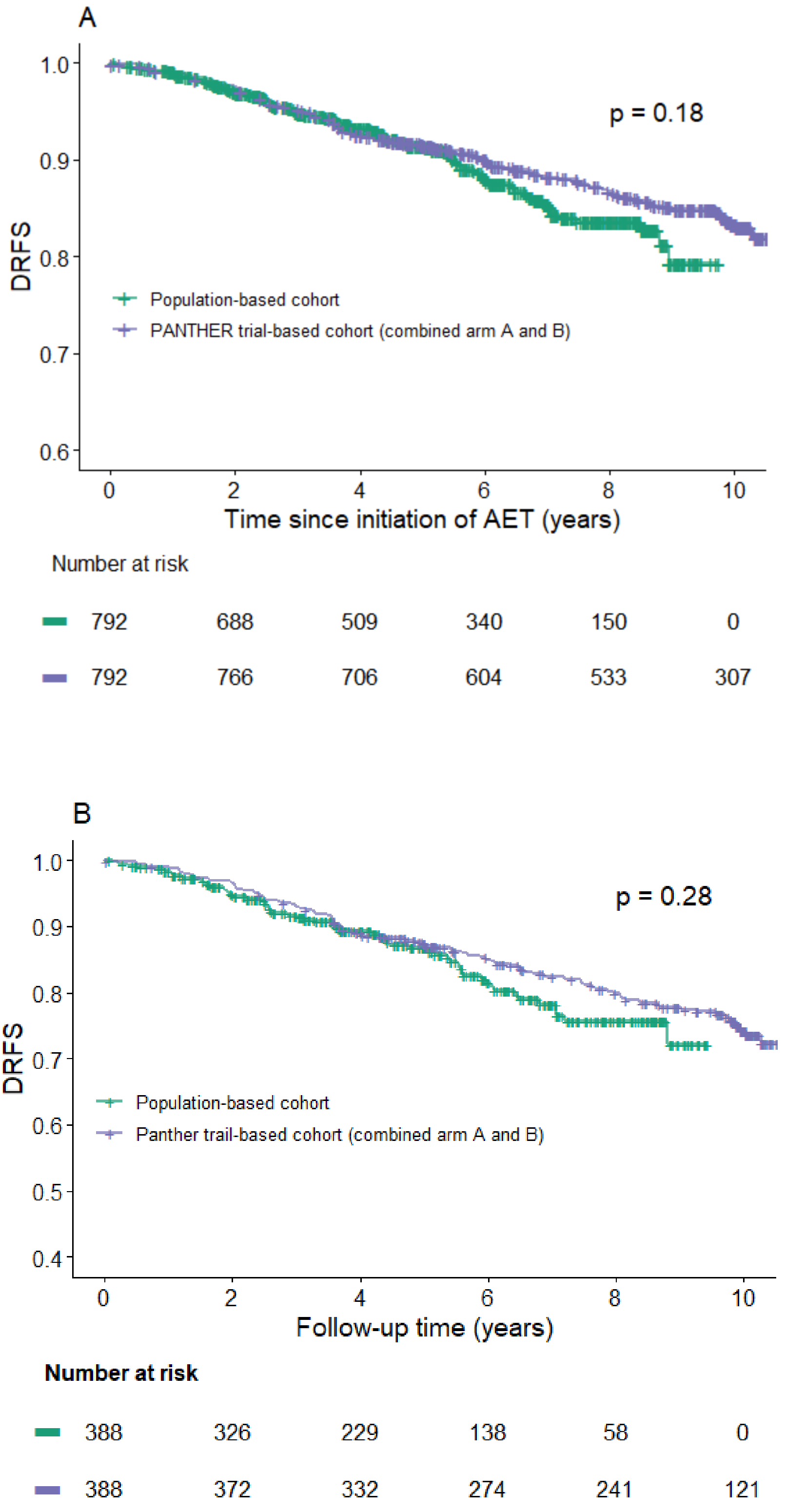

Supplement: Supplementary Tables and Figures [file mmc1.pdf]
