# Supplementary material for: Prevalence and prognosis of patients with breast cancer eligible for adjuvant abemaciclib or ribociclib: a nationwide population-based study
Source: Lancet Reg Health Eur. 2025 Sep 29;59:101471. doi: 10.1016/j.lanepe.2025.101471 (PMC12513154; doi:10.1016/j.lanepe.2025.101471)
Supplement: Statistical Analysis Plan [file mmc2.pdf]

<table>
<tr><td colspan="3">Statistical Analysis Plan
(tailored for the second part analysis comparing survival outcomes between trial-based participants and population-based cohorts, using regression standardization on pooled data)</td></tr>
<tr><td></td><td>Item</td><td>Note</td></tr>
<tr><td>1</td><td>Research Question and Objectives</td><td></td></tr>
<tr><td></td><td>Research question:
What is the difference in the 5- and 10-year cumulative incidence of distant relapse or death between patients treated under the PANTHER trial protocol (two arms) versus those treated under real-world routine care as observed in the population-based cohort, after adjusting for covariates?
Objective:
To estimate adjusted (standardized) cumulative incidence curves and compare the 5- and 10-year cumulative incidence of distant relapse or death in trial-based participants and a population-based cohort, after adjustment for baseline covariates.</td><td></td></tr>
<tr><td>2</td><td>Study Design and Data Sources</td><td></td></tr>
<tr><td></td><td>This is a comparative study using a subgroup of the original PANTHER-trial and a population-based cohort, with all individuals meeting NATALEE- and monarchE-inclusion criteria, respectively.
The relevant data of ER+HER2- BC patients involved in the PANTHER trial were prospectively retrieved from the original trial database as the population-based cohort with data retrieved from the registry database.
Data Sources were described in the main text (please refer to it for further details).</td><td></td></tr>
<tr><td>3</td><td>Outcome Definition</td><td></td></tr>
<tr><td></td><td>Distant relapse-free survival:
Time to distant relapse or all-cause mortality, defined from the initiation of adjuvant endocrine treatments to the earliest occurrence of distant relapse or death from any cause, whichever came first, with patients censored at the date of last follow-up.</td><td></td></tr>
<tr><td>4</td><td>Cohort Alignment and Variable Harmonization</td><td></td></tr>
<tr><td></td><td>To improve comparability between trail-based participants and population-based cohorts:
• The population-based cohort will be restricted/aligned to patients who would have been eligible for the PANTHER trial, such as age, tumor size, and nodal status. This alignment procedure further supports the positivity assumption required for valid modelling and standardization approaches (Hernán, M. A., & Robins, J. M. (2010). Causal inference).
• Baseline variable definitions will be harmonized between two cohorts (e.g., age, type of adjuvant endocrine therapy, nodal status, tumor size, and pathological grade categorized the same if necessary).
• Survival outcome definitions will also be harmonized, based on the updated standardized definitions for efficacy endpoints (STEEP version</td><td></td></tr>
</table>

	2.0).<br>• Time zero (or the date of starting follow-up) will be aligned to reflect the initiation of adjuvant endocrine therapies.<br>• Define the new variable of cohort indicator to represent distinct chemotherapy strategies in combination with adjuvant endocrine therapy.	
5	**Data pooling**	
	Following alignment on PANTHER eligibility criteria and harmonization of baseline variables, data from both PANTHER-trial participants and the population-based cohort will be pooled to form an analytic population representing NATALEE-eligible (or monarchE-eligible) patients. The pooled dataset will include aligned and harmonized baseline covariates, survival outcomes, and a cohort indicator to support comparative analyses.	
6	**Statistical Methods**	
	**Modelling approach used to adjustment for covariates**<br>A Cox proportional hazards model will be fit on the pooled data from both the PANTHER-trial and population-based cohorts, including a cohort indicator (representing distinct chemotherapy strategies in combination with adjuvant endocrine therapy), age, pathological grade, tumor size, nodal status, and type of adjuvant endocrine therapy.<br>**Standardization approach by Hernán (2010)**<br>First, based on the above fitted model, predicted survival probabilities will be estimated under each exposure category for all individuals and averaged over the covariate distribution of the registry cohort. Then, adjusted cumulative incidence curves and the corresponding risk differences at 5- and 10-year will be reported.	
7	**Handling of Missing Data**	
	Complete-case analysis results will be reported if the proportion of missing baseline covariates was less than or around 5%. Otherwise, a sensitivity analyses will be performed using multiple imputation methods, assuming missing at random. All imputed datasets will be analysed separately and then pooled using Rubin's rules.	
8	**Sensitivity Analysis**	
	A matching-based approach (e.g. propensity score or exact matching) will be conducted to assess robustness of results from model-based methods, focusing on comparing survival outcomes between matched PANTHER trial-based participants and the population-based individuals.	
9	**Software**	
	The above analyses may be conducted in R version 4.4.2 using the following common packages such as *survival*, *adjustedCurves*, *MatchIt*, *mice*, *survminer*, and *ggplot2*	
10	**Documentation**	

	This SAP was discussed and finalized before the initial analysis (January 2025) between the first author (Xingrong Liu, statistician) and the corresponding author (Alexios Matikas, the team leader). The relevant analysis procedures were not influenced by the results.	
